# Supplementary material for: Content-rich biological network constructed by mining PubMed abstracts
Source: BMC Bioinformatics. 2004 Oct 8;5:147. doi: 10.1186/1471-2105-5-147 (PMC528731; doi:10.1186/1471-2105-5-147)
Supplement: Additional File 2 — The original results of the above study (non-essential files are deleted to keep the file size under the limit set by BMC bioinformatics). [file 1471-2105-5-147-S2.bz2 › chilibotAdditionalFile2/dip10/232ID7931156E751/html/UL9_UL8.html]

 


 **UL9** and **UL8** 
  
Found 20 abstracts in PubMed, retrieved 10.  
 

 What does Google say? 
 PDF only 
| .edu only 

---

**Interactive relationship** (e.g. stimulation, inhibition, etc)

**Neutral relationship**- Analysis of transcripts and time course studies demonstrated that the  **UL8**  protein is translated from a delayed early expressed 3.1 kb polyadenylated mRNA which initiates within the  **UL9**  ORF.  Ref: 9460934 J Gen Virol, 1998
- A truncated protein, consisting of the C terminal 487 amino acids of OBP  [ **UL9** ] , termed OBPC, is the product of the HSV  **UL8** .5 gene and binds to origin sequences, although OBPC s role in HSV DNA replication is not yet clear.  Ref: 11435559 J Virol, 2001

**Non-interactive relationship** (e.g. studied together, co-existance, homology, etc.)

- Homologs of essential HSV 1 replication proteins,  **UL8**  and  **UL9** , were also identified.  Ref: 9155884 Arch Virol, 1997
- Within this region eight open reading frames were identified whose deduced amino acid sequences exhibited homology to the UL6, UL7,  **UL8** ,  **UL8** .5,  **UL9** , UL10, UL11, and UL12 protein products of herpes simplex virus type 1.  Ref: 9155870 Arch Virol, 1997
- Since this pattern can form in cells infected with mutants which are defective in UL5,  **UL8** ,  **UL9** , or UL52 in the presence of polymerase inhibitors C.  Ref: 9151871 J Virol, 1997
- Transcriptional analyses indicated presence of 3 coterminal mRNAs for genes  **UL8** ,  **UL8** .5, and  **UL9**  as well as for genes UL6 and UL7, respectively, while UL10 was represented by a very abundant unique transcript.  Ref: 9155870 Arch Virol, 1997
- The results shown in this paper are consistent with the model that the five proteins, ICP8, UL5,  **UL8** ,  **UL9** , and UL52, form a scaffold.  Ref: 12634381 J Virol, 2003
- The ordered assembly of the herpes simplex virus HSV type 1 replication apparatus leading to replication compartments likely involves the initial assembly of five viral replication proteins, ICP8,  **UL9** , and the heterotrimeric helicase primase complex UL5  **UL8**  UL52, into replication foci.  Ref: 12634381 J Virol, 2003
- The observed effect on herpes simplex virus DNA replication was not caused by a decreased transcription of replication genes inasmuch as the levels of  **UL8** ,  **UL9** , UL29, and UL30 rmRNAs were unaffected by the drug.  Ref: 8676478 J Virol, 1996
- The assembly of complete HSV replication compartments and incorporation of BrdU were both abolished by treatment with phosphonoacetic acid and by omission of any one of the seven viral replication proteins, UL5,  **UL8** ,  **UL9** , UL42, UL52, SSB, and Pol, that are essential for viral DNA replication.  Ref: 9060678 J Virol, 1997
